# Evaluation of the Cytotoxicity of *Satureja spicigera* and Its Main Compounds

**DOI:** 10.1100/2012/203861

**Published:** 2012-04-24

**Authors:** Ahmad Reza Gohari, Seyed Nasser Ostad, Fahimeh Moradi-Afrapoli, Maryam Malmir, Shohreh Tavajohi, Hassan Akbari, Soodabeh Saeidnia

**Affiliations:** ^1^Medicinal Plants Research Center, Faculty of Pharmacy, Tehran University of Medical Sciences, P.O. Box 14155-6451, Tehran 14176 14411, Iran; ^2^Department of Toxicology, Faculty of Pharmacy, Tehran University of Medical Sciences, Tehran 14176 14411, Iran

## Abstract

*Satureja spicigera* (Lamiaceae) grows wildly in Northwest of Iran. In this study, bioassay-guided isolation and identification of the main compounds has been reported using various chromatographic methods and comparison of their spectral data with those reported in the literature. Brine shrimp lethality and four cancerous cell lines HT29/219, Caco_2_, NIH-3T3, and T47D were used for cytotoxicity evaluations. From the aerial parts of *S. spicigera*, nine known compounds including two flavanones, 5,7,3′,5′-tetrahydroxy flavanone (**8**) and 5,4′-dihydroxy-3′-methoxyflavanone-7-(6′′-O-*α*-L-rhamnopyranosyl)-*β*-D-glucopyranoside (**9**), one dihydrochalcone, nubigenol (**7**), together with thymoquinone (**1**), thymol (**2**), carvacrol (**3**), *β*-sitosterol (**4**), ursolic acid (**5**) and oleanolic acid (**6**) were identified. Among the isolated chalcone and flavanones, compound **8** was effective against *Artemia salina* larva (LC_50_= 2 *μ*g/mL) and only the compound **9** demonstrated IC_50_ value of 98.7 *μ*g/mL on the T47D (human, breast, ductal carcinoma). Other compounds did not show significant inhibition of the cell growth.

## 1. Introduction

The genus *Satureja* (Lamiaceae) has 13 species in Iran and is called Marzeh. One of these species is *S. spicigera *that grows wildly in Northwest of Iran [1, 2]. Recent phytochemical studies indicated the presence of flavanones (naringenin, aromadendrin, eriodictyol, taxifolin, and flavanone trimethyl ether) and flavones (apigenin, luteolin, diosmetin, genkwanin, ladanein, thymosin, thymonin, cirsimaritin, and xanthomicrol) in some species of *Satureja *[3–5].

So far, antimicrobial [6], spasmoltic [7], anti-HIV [8], antiviral [9], antioxidant [10] and cytotoxic activities [11, 12] have been reported from several species of this genus. Volatile composition of some Iranian species of *Satureja* has been investigated [13]. Recently, we have reported the chemical composition of the volatile oil of *S. spicigera *[14]. The oil of *S. spicigera* was rich in monoterpenes (89.9%) with thymol (37.3%) as the major compound [14]. Comparison of some *Satureja* species by phylogenetic and chemotaxonomic analysis showed that the genetic distance between *S. spicigera* and *S. mutica* is close [15]. Antibacterial activity of the oil of *S. spicigera *has been reported against *B. subtilis*, *S. aureus, E. coli, *and* K. pneumonia* [16]. Also, we reported the trypanocidal activities of the several extracts of *S. spicigera *and other species of this genus [17–19]. A literature survey has shown that the phytochemical constituents of *S. spicigera* were not previously published. Therefore, we aim to report the isolation, structural elucidation, and cytotoxicity of the main constituents of *S. spicigera *for the first time.

## 2. Material and Methods

### 2.1. General Procedures

^1^H- and ^13^C-NMR spectra were measured on a Bruker Avance TM 500 DRX (500 MHz for ^1^H and 125 MHz for ^13^C) spectrometer with tetramethylsilane as an internal standard, and chemical shifts are given in *δ* (ppm). HR-ESITOFMS were obtained on a Bruker MicroTOFII mass spectrometer. The FT-IR spectra were recorded on a Nicolet 550 instrument. Silica gel 60F_254_ precoated plates (Merck TM) were used for TLC. The spots were detected by spraying anisaldehyde-H_2_SO_4_ reagent followed by heating (120°C for 5 minuets).

### 2.2. Plant Material

Aerial parts of *Satureja spicigera *(C. Koch) Boiss, at flowering stage, were collected from Astara in the Northwest of Iran, in September 2004. Voucher specimen (78410 TARI) was deposited at the Herbarium of the Institute of Forests and Rangelands Researches. Plant specimen was identified by Dr. Vali-allah Mozaffarian from the same institute.

### 2.3. Extraction, Isolation, and Structural Elucidation

The flowered aerial parts of *S. spicigera* (1 kg) was cut into small pieces and extracted with ethyl acetate and methanol, consequently, at room temperature. The ethyl acetate extract (23 g) was subjected to silica gel column chromatography (CC) with hexane : CHCl_3_ (8 : 2), CHCl_3_ : AcOEt (2 : 8), and AcOEt as eluent to give six fractions (A–F). The fraction C (7 g) was submitted to silica gel CC with hexane : AcOEt (19 : 1) to obtain five fractions C_1_–C_5_. The fraction C_2_ (97 mg) was chromatographed on sephadex LH_20_ with AcOEt : MeOH (3 : 1) to result compound **1 **(14 mg). The fraction C_3_ (1 g) was subjected to CC with hexane : AcOEt (9 : 1) to give four fractions C_31_–C_34_. The compounds **2** (35 mg) and **3 **(15 mg) were given from the fractions C_32_ and C_33_, respectively, using sephadex LH_20_ eluted with AcOEt : MeOH (3 : 1). The fraction C_5_ (400 mg) was submitted to silica gel CC with hexane : AcOEt (9 : 1) to obtain compound **4** (26 mg).

The MeOH extract (70 g) was successively subjected to silica gel CC with hexane : AcOEt (8 : 2), CHCl_3_ : AcOEt (8 : 2), AcOEt, and MeOH as eluent to give eight fractions (M_1_–M_8_). Fraction M_3_ (5.3 g) was fractionated on CC with CHCl_3_ : AcOEt (8 : 2) to yield compounds **5** (320 mg) and **6** (45 mg). The fraction M_4_ (690 mg) was chromatographed on sephadex LH_20_ with MeOH to result M_41_–M_43_. Fraction M_43_ (44 mg) was purified on sephadex LH_20_ with MeOH to yield compound **7 **(15 mg). Compound **8** (26 mg) was yielded from fraction M_5_ (1.9 g) using twice sephadex LH_20_ with MeOH. The fraction M_8_ (9 g) was submitted to reverse- phase (C_8_) CC with aqueous MeOH (30%, 50%, and 100%) to give 7 fractions (M_81_–M_87_). Compound **9** (90 mg) was obtained from fraction M_87_ (3.3 g) using sephadex LH_20_ with MeOH.

### 2.4. Brine Shrimp Lethality Assay (BSA)

Gohari et al. reported the method which was adopted to study the cytotoxic activity of the compounds [11]. Water life brand brine shrimp (*Artemia salina*) eggs were purchased from the Shalat Center (Tehran). The eggs were hatched in a flask containing 300 mL artificial seawater made by dissolving distilled water. The flask was well aerated with the aid of an air pump and kept in a water bath at 29-30°C. A bright light was left on. The nauplii hatched within 48 h. The extracts and pure compounds were dissolved in normal saline. Different concentrations were obtained by serial dilution. Solution of each concentration (500 *μ*L) was transferred into clean 24-well plates via a pipette, and aerated seawater having 10–20 nauplii (500 *μ*L) was added. A check count was performed and the number alive noted after 24 h. The mortality end point of the bioassay was determined as the absence of controlled forward motion during 30 seconds of observation. The controls used were seawater and berberine hydrochloride (LC_50_ = 26 *μ*g/mL). Lethality percentage was determined and LC_50_ calculated based on probit analysis with 95% of confidence interval [11].

### 2.5. Cell Cultures and Cytotoxicity Assay

Four cancerous cell lines HT29/219 (human, colon, epithelial-like, carcinoma), Caco_2_ (human, colon, adenocarcinoma), NIH-3T3 (Swiss NIH mouse, embryo fibroblast), and T47D (human, breast, ductal carcinoma) were purchased from the Pasteur Institute, Tehran, Iran. The cells were maintained in RPMI 1640, supplemented with 10% fetal bovine serum, 0.28 units/mL insulin, 100 *μ*g/mL streptomycin, 100 units/mL penicillin, and 0.3 mg/mL glutamine. The cells were grown at 37°C in a humidified atmosphere of 5% CO_2_, in air.

The cytotoxicity of the compounds isolated from *S. spicigera* was assayed using the MTT cytotoxicity assay. The cells (3 × 10^ 4^) were plated in 500 *μ*L of medium/well in 48-well plates (NUNC Cell Culture Flasks, Denmark). After an overnight incubation at 37°C, in 5% CO_2_, and a humidified atmosphere, the samples were added to the cells to a final concentration of 500 *μ*g/mL. “Methotrexate” (positive control) and pure compounds were examined at concentrations ranging from 5, 10, 20, 40, 80, and 100 *μ*g/mL. The plates were incubated at 37°C, in 5% CO_2_, humidified atmosphere, for 48 hours. After 48 hours, 50 *μ*L of 5 mg/mL MTT (dissolved in PBS) was added per well. After three hours of incubation, the MTT solution was removed and the cells were washed with 100 *μ*L of PBS, twice. One hundred and fifty microlitres of DMSO was added per well, to solubilize the formazan crystals. The optical densities of the wells were then measured at 570 nm (690 nm reference wavelength). By referring to the control (medium with DMSO), the cell survival was assessed [20].

## 3. Results

**Figure 1:**
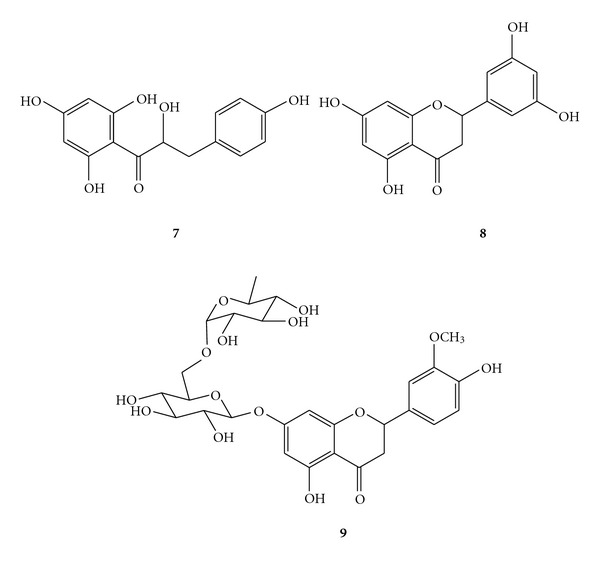
Chemical structures of the isolated dihydrochalcone and flavanones from *Satureja spicigera*.

Dried aerial parts of *S. spicigera*, collected during full flowering stage, were successively extracted with ethyl acetate and methanol. These extracts were concentrated and examined with brine shrimp lethality assay (BSA). Both extracts showed cytotoxic activity against *Artemia salina *larvae, so were used for further isolation on silica gel and sephadex column chromatography to obtain compounds **1**–**9** (Figure 1). Isolated compounds, **1**–**7**, were identified as thymoquinone (**1**), thymol (**2**), carvacrol (**3**), *β*-sitosterol (**4**), ursolic acid (**5**), oleanolic acid (**6**), and nubigenol (**7**) by comparison of their spectral data with those reported in literature [11, 21–23]. The results have a good agreement with references. Full assignments of the compounds **8** and **9**, performed by 2D-NMR and HR-ESITOFMS techniques, have been reported in this paper, because they have not been found in the literature review [24, 25].

The results obtained from BSA showed that the compound **8** (LC_50_ = 2 *μ*g/mL) was effective against larvae of* Artemia salina.* This is a toxic compound compared to berberine hydrochloride (LC_50_ = 26 *μ*g/mL) which was used as a positive control. Among the three various compounds of *S. spicigera*, the compound **9 **demonstrated IC_50_ value of 98.7 *μ*g/mL on the T47D (human, breast, ductal carcinoma) (*P* < 0.01). The other compounds did not show significant inhibition of the cell growth or proliferation, even compound **8 **which showed toxicity against brine shrimp larvae (Table 3).

***5,7,3′,5′-Tetrahydroxy Flavanone *(8)**

Pale yellow crystal; melting point 264–268°C; IR (CHCl_3_) **ν**_max_ 3353 (O–H), 2974, 2924, 2855 (C–H), 1627, 1598 (C=O), 1435, 1114, 695 cm^−1^; HR-ESITOFMS *m/z *311.0526 [M + Na]^+^ (calcd for C_15_H_12_O_6_Na, 311.0532). NMR spectra are shown in Table 1.

***5,4′-Dihydroxy-3′-methoxyflavanone-7-(6′′-O-*α*-L-rhamnopyranosyl)-*β*-D-glucopyranoside *(9)**

White amorphous crystal; melting point 256°C; HR-ESITOFMS *m/z* 633.1790 [M + Na]^+^ (calcd for C_28_H_34_O_15_Na, 633.1795). NMR data is shown in Table 2.

## Figures and Tables

**Table 1:** NMR spectra of the compound **8** in DMSO-*d*_6_.

No.	^13^C-NMR	^1^H-NMR	HMBC
2	78.4	5.37 (*dd*, *J *= 12.4, 2.9 Hz, 1H)	H-3b, H-2′ or H-6′
3	42.1	2.69 (*dd*, *J *= 17.0, 2.9 Hz, 1H)	
		3.18 (*dd*, *J *= 17.0, 12.5 Hz, 1H)	
4	196.2		H-3a, H3b
5	162.9		H-6
6	95.8	5.88 (*d*, *J *= 1.2 Hz, 1H)	5-OH
7	166.7		H-6 or H-8
8	95.0	5.88 (*d*, *J *= 1.2 Hz, 1H)	
9	163.4		H-8, 5-OH
10	101.7		H-6 or H-8, 5-OH
1′	129.4		H-3b, H-2′ or H-6′
2′	117.9	6.75 (*brs*, 1H)	H-4′
3′	145.7		H-4′
4′	114.3	6.88 (*brs*, 1H)	H-2′ or H-6′
5′	145.2		H-4′, H-2′ or H-6′
6′	115.3	6.75 (*brs*, 1H)	
5-OH		12.14 (*s*, 1H)	
7-OH		10.76 (*s*, 1H)	
3′-OH		9.00 (*s*, 1H)	
5′-OH		9.05 (*s*, 1H)	

**Table 2:** NMR spectra of the compound **9** in DMSO-*d*_6_.

No.	^13^C-NMR	^1^H-NMR	HMBC
2	78.4	5.50 (*dd*, *J *= 12.5, 3.0 Hz, 1H)	H-3b, H-2′
3	42.1	2.77 (*dd*, *J *= 17.5, 3.2 Hz, 1H)	
		3.14 (*m*, 1H)	
4	197.1		H-3a, H-3b
5	162.5		H-6
6	96.4	6.13 (*d*, *J *= 2.5 Hz, 1H)	H-8
7	165.2		H-6, H-8
8	95.6	6.14 (*d*, *J *= 2.5 Hz, 1H)	
9	163.1		H-8
10	103.4		H-6
1′	131.9		H-2, H-6′
2′	114.2	6.94 (*m*, 1H)	
3′	146.5		H-5′, OCH_3_
4′	148.0		
5′	112.1	6.94 (*m*, 1H)	
6′	118.0	6.89 (*dd*, *J* = 8.0, 2.0 Hz, 1H)	H-2, H-2′
–OCH_3_	55.7	3.79 (*s*, 3H)	
5-OH		12.08	
Glc-1′′	99.5	4.97 (*d*, *J* = 7.5 Hz, 1H)	
2′′	73.0	3.21 (*m*, 1H)	
3′′	76.3	3.26 (*m*, 1H)	
4′′	69.6	3.15 (*m*, 1H)	
5′′	75.5	3.54 (*m*, 1H)	
6′′	66.1	3.37 (*m*, 1H)	H-1′′′
		3.80 (*m*, 1H)	
Rha-1′′′	100.6	4.51 (*d*, *J* = 1.0 Hz, 1H)	
2′′′	70.7	3.62 (*m*, 1H)	H-1′′′
3′′′	70.3	3.42 (*m*, 1H)	
4′′′	72.1	3.17 (*m*, 1H)	
5′′′	68.4	3.42 (*m*, 1H)	
6′′′	17.9	1.08 (*d*, *J* = 6.0 Hz, 3H)	H-1′′′

**Table 3:** The effects of dihydrochalcone and flavanones isolated from *S. spicigera* on the viability of various cell lines using MTT assay.

Sample	Cell lines^a^ (MTT assay)			
	T47D	Caco-2	HT-29	NIH 3T3
**7**	132.0	143.0	>150	112.0
**8**	>150	>150	>150	100.7
**9**	98.7	>150	>150	140.4

^a^Results are expressed as IC_50_ values (*μ*g/mL), Key to cell Lines employed: HT-29 and Caco-2 (colon adenocarcinoma); T47D (breast carcinoma); NIH 3T3 (Swiss embryo fibroblast).
